# Supplementary material for: CD147 mediates intrahepatic leukocyte aggregation and determines the extent of liver injury
Source: PLoS One. 2019 Jul 10;14(7):e0215557. doi: 10.1371/journal.pone.0215557 (PMC6619953; doi:10.1371/journal.pone.0215557)
Supplement: S1 Table — Start and end mouse body weights for C57Bl/6 and Balb/c mice following hepatotoxin treatment and/or antibody intervention. Data are expressed as mean ± SEM. Mann-Whitney test was performed to assess significance from control where *** p<0.001 vs H2O Control, **** p<0.0001 vs H2O Control. H2O control = H2O only, PO control = PO only, CCl4 injury = CCl4 injury alone, TAA = TAA injury alone, αCD147 = anti-CD147 mAb in CCl4 injury, IgG2a = Isotype mAb control in CCl4 injury. (DOCX) [file pone.0215557.s002.docx]

| **S1 Table. Changes in mouse weights following hepatotoxin treatment or antibody intervention** | | | | | |
| --- | --- | --- | --- | --- | --- |
| Mouse Strain | Treatment | Number of mice per group | Timepoint | Start Weight (grams) | End Weight (grams) |
| C57Bl/6 | H_2_O Control | 9 | 4 weeks | 21.18 ± 0.35 | 27.59 ± 0.49 |
|  | TAA | 9 | 4 weeks | 22.02 ± 0.33 | 22.52 ± 0.24 **** |
|  | H_2_O Control | 12 | 8 weeks | 21.93 ± 0.32 | 31.87 ± 0.91 |
|  | TAA | 12 | 8 weeks | 22.72 ± 0.33 | 26.28 ± 0.95*** |
|  | H_2_O Control | 5 | 20 weeks | 25.28 ± 0.48 | 32.5 ± 0.74 |
|  | TAA | 9 | 20 weeks | 24.08 ± 0.41 | 32.43 ± 0.48 |
|  | PO control | 6 | 1 day | 23.02 ± 0.47 | 24.24 ± 0.56 |
|  | CCl_4_ | 6 | 1 day | 23.81 ± 0.38 | 24.57 ± 0.33 |
|  | PO Control | 8 | 4 weeks | 22.61 ± 0.71 | 25.14 ± 0.75 |
|  | CCl_4_ | 8 | 4 weeks | 23.41 ± 0.33 | 25.96 ± 0.31 |
|  | CCl_4_ + αCD147 | 8 | 4 weeks | 23.02 ± 0.80 | 25.08 ± 0.82 |
|  | CCl_4_ + IgG2a | 8 | 4 weeks | 23.04 ± 0.37 | 24.98 ± 0.32 |
| BALB/c | PO Control | 8 | 4 weeks | 22.89 ± 0.68 | 23.91 ± 0.62 |
|  | CCl_4_ | 6 | 4 weeks | 22.97 ± 0.64 | 23.65 ± 0.87 |
|  | CCl_4_ + αCD147 | 5 | 4 weeks | 23.30 ± 0.37 | 23.22 ± 0.54 |
|  | CCl_4_ + IgG2a | 8 | 4 weeks | 23.00 ± 0.22 | 24.31 ± 0.35 |
|  | Expressed as mean ± SEM  Mann-Whitney t test where *** p<0.001 vs Control, **** p<0.0001 vs Control | | | | |
